# TaijiGNN: A New Cycle-Consistent Generative Neural Network for High-Quality Bidirectional Transformation between RGB and Multispectral Domains

**DOI:** 10.3390/s21165394

**Published:** 2021-08-10

**Authors:** Xu Liu, Abdelouahed Gherbi, Wubin Li, Zhenzhou Wei, Mohamed Cheriet

**Affiliations:** 1 Synchromedia Laboratory, École de Technologie Supérieure (ÉTS), University of Québec, Montréal, QC H3C 1K3, Canada; Abdelouahed.Gherbi@etsmtl.ca (A.G.); Mohamed.Cheriet@etsmtl.ca (M.C.); 2 Ericsson Research, Montréal, QC H3C 1K3, Canada; wubin.li@ericsson.com; 3 Department of Electrical and Computer Engineering, McGill University, Montréal, QC H3C 1K3, Canada; zhenzhou.wei@mail.mcgill.ca

**Keywords:** spectral super-resolution, multilayer perceptron, cycle neural network, CycleGAN, multispectral image, hyperspectral image, RGB image, image translation, image processing, color vision, computer vision

## Abstract

Since multispectral images (MSIs) and RGB images (RGBs) have significantly different definitions and severely imbalanced information entropies, the spectrum transformation between them, especially reconstructing MSIs from RGBs, is a big challenge. We propose a new approach, the Taiji Generative Neural Network (TaijiGNN), to address the above-mentioned problems. TaijiGNN consists of two generators, G_MSI, and G_RGB. These two generators establish two cycles by connecting one generator’s output with the other’s input. One cycle translates the RGBs into the MSIs and converts the MSIs back to the RGBs. The other cycle does the reverse. The cycles can turn the problem of comparing two different domain images into comparing the same domain images. In the same domain, there are neither different domain definition problems nor severely underconstrained challenges, such as reconstructing MSIs from RGBs. Moreover, according to several investigations and validations, we effectively designed a multilayer perceptron neural network (MLP) to substitute the convolutional neural network (CNN) when implementing the generators to make them simple and high performance. Furthermore, we cut off the two traditional CycleGAN’s identity losses to fit the spectral image translation. We also added two consistent losses of comparing paired images to improve the two generators’ training effectiveness. In addition, during the training process, similar to the ancient Chinese philosophy Taiji’s polarity Yang and polarity Yin, the two generators update their neural network parameters by interacting with and complementing each other until they all converge and the system reaches a dynamic balance. Furthermore, several qualitative and quantitative experiments were conducted on the two classical datasets, CAVE and ICVL, to evaluate the performance of our proposed approach. Promising results were obtained with a well-designed simplistic MLP requiring a minimal amount of training data. Specifically, in the CAVE dataset, to achieve comparable state-of-the-art results, we only need half of the dataset for training; for the ICVL dataset, we used only one-fifth of the dataset to train the model, but obtained state-of-the-art results.

## 1. Introduction

### 1.1. The Background

Different wavelength lights have various propagating, reflecting, and refracting properties. Multispectral images (MSIs) use distinct bands to record different wavelength properties and contain affluent spectral information [1]. With the rich spectral information, MSIs can help to solve problems that RGBs can not, such as MSIs for the retinal oximetry measurements in four diseased eyes [2], the detection of adulteration with different grains in wheat products [3], the non-contact analysis of forensic traces [4], and the visualization of latent traces in crime scenes [5].

Although MSIs have a broad application scope, acquiring them is a time-wasting, complicated and expensive process. Tens or hundreds of MSI bands have to be taken one by one, which consumes a significant amount of time and storage space resulting in big, complicated, and expensive multispectral apparatuses.

On the other hand, RGB images (RGBs) have only three channels: blue, green, and red. Additionally, they form other colors by composing the three primary colors with different ratios, and their formation principle is different from that for spectral images. Moreover, RGBs can be quickly and cheaply obtained by ordinary consumer cameras, such as mobile phone cameras. Although the RGB images can give us a good color impression about the objects or the scenarios, they contain scarce spectral information and have many restrictions.

In sum, RGBs and MSIs have their pros and cons. It would be meaningful to create a model that can perform bi-directional translations between RGBs and MSIs. However, there are two significant challenges to be solved first.

The first challenge is that RGBs and MSIs have different definitions and formation mechanisms. Although we can synthesize RGBs from MSIs with color matching functions, they can work under limited circumstances, as long as there is sufficient visible light (380 nm–700 nm) spectral information. In other circumstances, when we only have several bands, such as near-infrared (NIR), infrared (IR), and ultraviolet (UV), it is difficult to use the color matching function to synthesize a real RGB-like image [6]. Moreover, the color matching functions are often too complex to accelerate in a parallel manner. Furthermore, no color matching function can synthesize MSIs from RGBs directly [7].

The second challenge is that the spectral resolution of MSIs is much higher than that of the RGBs’. MSIs often contain much more information than RGBs. One RGB composition may map to many kinds of MSI composition named metamerism [8]. Reconstructing MSIs from RGBs is a severely underconstrained problem.

Most previous related works meet a bottleneck when trying to improve reconstructing MSI performance. As they treat the two reconstruction processes, RGBs->MSIs (converting RGBs to MSIs) and MSIs->RGBs (converting MSIs to RGBs) as two separate processes without any relations. They only concentrated on rebuilding MSIs from RGBs and ignored the process of MSIs->RGBs, since they thought that MSIs->RGBs is obvious and no help to reconstruct MSIs from RGBs. Thus, they did not discover the link between the two processes. However, the two processes have a complementary relation. If we treat them separately, we may lose a large amount of valuable information, which would lead to poor reconstruction and miss the opportunity to solve the above two problems.

Our approach TaijGNN is inspired by the ancient Chinese philosophy “Taiji” [9] and Cycle Generative Adversarial Network (CycleGAN) [10] and merged with some of our recent considerations. It has two in-out ports and two generators. One port is for inputting or outputting RGBs. The other port is for MSIs. Generator $G_{MSI}$ converts the RGBs into MSIs. Generator $G_{RGB}$ converts the MSIs into RGBs. Moreover, the two ports and two generators are formed into two cycles, (1) and (2).

(1) $$\underset{\left(Original\right)}{RGB}\overset{G_{MSI}}{\to }\underset{\left(Generated\right)}{MSI^{\prime }}\overset{G_{RGB}}{\to }\underset{\left(CycleGenerated\right)}{RGB\prime \prime }$$

(2) $$\underset{\left(Original\right)}{MSI}\overset{G_{RGB}}{\to }\underset{\left(Generated\right)}{RGB^{\prime }}\overset{G_{MSI}}{\to }\underset{\left(CycleGenerated\right)}{MSI\prime \prime }$$

Since the most important fact causing the above two challenges is that RGBs and MSIs belong to two distinct domains, the conversions between them would be similar to the conversions between images and labels. The two cycles, (1) and (2), ingeniously covert the problem of two different domain conversion into the same domain conversion. As we know, it is not difficult to convert one image to itself in the same domain. In this way, the two challenges can be solved naturally.

Moreover, most researchers believe that complex deep neural networks would improve the models’ performance, and the more complex the neural networks are, the better effect they will have. Therefore, their approaches have often adopted tens or hundreds of convolutional neural network (CNN) layers with hundreds of residual network layers, which leads to a long computing time and high power consumption [11,12,13]. According to our thorough investigations and experiments, normal 2D convolutional operations scarcely improve the spectrum reconstruction performance and make the generated images blurry. Therefore, in our generators, we remove the traditional convolution layers and mainly adopt multilayer perceptron (MLP) architecture. The following experiments (Section 4) prove that TaijiGNN based on MLP has a higher performance than the related works based on CNN.

Additionally, unlike traditional CycleGAN, in TaijiGNN, we also compare the generated image with the paired ground truth image to enhance the generator’s training performance. For example, in Cycle (1), when we used Generator $G_{MSI}$ to convert the RGB into the MSI, we could calculate their mean absolute error (MAE) to update Generator $G_{MSI}$. On the contrary, the traditional CycleGAN can only use adversary networks to determine if the generated images belong to the domain due to lacking the paired dataset. Moreover, we found that the indirect adversary network learning efficiency is much lower than that of direct supervised learning. Therefore, TaijiGNN eliminates the adversary network in its architecture, dramatically increasing the training speed and the model’s performance.

**Figure 1:**
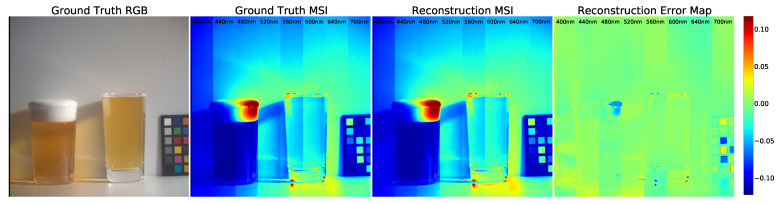
An example of reconstructing a multispectral image from an RGB image.

Figure 1 shows TaijiGNN’s reconstruction performance. We used the trained model to reconstruct an MSI from an RGB and integrated eight selected bands into one image. Additionally, the reconstruction error map presents the degree of dissimilarity. More details are provided in the following sections.

### 1.2. The Contributions

Our contributions are mainly in the following three aspects:

- We proposed a new neural network, TaijiGNN (Taiji Generative Neural Network), which takes advantage of cycled neural network architecture to convert the problem of comparing two different domain images into comparing the same domain images, which can supply a solid theory foundation to solve the two distinct domain image translation problems.
- We effectively designed a multilayer perceptron neural network to substitute the convolutional neural network when implementing the generators. Additionally, we used solid experiments to prove that our MLP generators perform better than the traditional generators based on CNN during spectral super-resolution.
- We innovatively eliminated the traditional CycleGAN’s identity loss to tackle the different domain images with different dimensions and induced two consistency losses in TaijiGNN to enhance the training performance.

The remaining parts of the paper are organized as follows: First, we demonstrate several related studies in Section 2. Then, the proposed approach is elaborated in Section 3. Next, in Section 4, the qualitative and quantitative experiments and comparisons conducted to assess our approach are described. Moreover, we discuss the proposed approach’s limitations in Section 5. Furthermore, in the last Section 6, a summary and future work are introduced. In Appendix A, we provide the details of TaijiGNN’s training and inferencing performance information.

## 2. Related Work

As described in Section 1, direct spectrum translations between MSIs and RGBs have many benefits. Many researchers have contributed to this area.

In 2014, Nguyen et al. combined whited balanced RGB images and the radial basis function (RBF) network to reconstruct multispectral images. The performance of their approach is acceptable when the spectrum of illumination and reflectance is flat. Nevertheless, when the spectrum becomes spiked, their method failed to maintain a good performance. Their work also has some other restrictions, such as that all scenes are under the same illumination [14].

Two years later, in 2016, with the RGB projection and the sparse dictionary of multispectral signatures, Arad et al. successfully improved the conversion of MSIs from RGBs. They obtained top results at that time. However, their method must sample some information from every RGB image and MSI to make the multispectral image prior to reconstructing the MSIs, narrowing their applied fields. Additionally, their MSI recovery quality heavily depends on the scenes [15].

The previous methods were overly reliant on all kinds of prerequisites, such as the environmental factors or the device’s specifications. Once the prerequisites change, they have to rebuild their models according to the new prerequisites. On the contrary, deep learning approaches are data-driven. Once we finish the deep learning network architecture design, it is not necessary to redesign the whole network even when the scenes change. Re-training the model with the new dataset is the only necessary action. Therefore, an increasing number of spectrum conversion approaches take advantage of this recent technology.

Choi et al. leveraged an autoencoder to restore MSIs from RGBs in 2017. Their approach avoided the trade-off between spectral accuracy and spatial resolution. However, it only involves restoring hyperspectral images from compressive spectral images, but not reconstructing the MSIs from the RGBs [16]. In the same year, Zhiwei et al. suggested a unified deep learning framework named HSCNN, reconstructing MSIs from RGBs. They used simple interpolation to upsample RGBs and leveraged the paired upsampled RGBs and MSIs to train the CNN model. Furthermore, they declared that their approach surpassed all previous approaches [17].

In 2018, Zhan et al. upgraded HSCNN’s RGB upsampling process from a handcrafted operation to a simple CNN. They named their new approach “HSCNN+”. Additionally, they replaced the HSCNN’s regular convolution layers with residual blocks or dense blocks. Additionally, they gave the name “HSCNN-R” to the network with residual blocks and named the network with the dense blocks “HSCNN-D” [18]. Moreover, HSCNN-D was the winner, and HSCNN-R achieved second place in the NTIRE 2018 Spectral Reconstruction Challenge [19].

In the same year, 2018, Berk et al. successfully produced a multispectral image from an RGB image in the wild. They first assessed the sensitivity function from an RGB image, and then proposed a classifier network to predict which function can be used to constitute the image. Finally, they built a reconstruction model to convert the RGB image to the multispectral image [20]. They obtained the seventh place in the NTIRE 2018 Spectral Reconstruction Challenge, and they declared that their approach has the fewest layers and the fastest speed.

Moreover, Kin et al. demonstrated how to leverage the conditional GAN to conduct bi-directional spectrum translation between multispectral images and RGB images in the 2019 Conference on Computer Vision and Pattern Recognition. Although they made some progress in the spectrum reconstruction performance, they did not give a reasonable explanation of how to supplement the lost multispectral information [21].

Recently, in 2020, in the “NTIRE Challenge on Spectral Reconstruction from an RGB Image”, several new approaches emerged [22]. The champion, Jiaojiao et al., suggested using the adaptive weighted attention network to restore the multispectral image from the RGB image. Their neural network mainly consists of several dual-residual attention blocks. Additionally, they obtained the best result in the “Clean” dataset and ranked third in the “Real World” dataset [23].

Although the previous related papers have achieved some progress in reconstructing spectral images, no one clearly explained how their approaches solve the underconstrained spectrum translation in theory. Moreover, most related works neglect the differences between spectrum reconstruction problems and other image processing, try to use traditional stacking and design complex CNN layers to solve the problem. However, since traditional CNNs mainly perform convolutions on the 2D surface, except addition operations, there are no convolutional operations on the spectral dimension. The relation among different spectral bands may be overlooked. Additionally, deep neural networks may improve the generator’s performance, but too many neural layers may cause a series of gradient vanishing, overfitting, and long executing time problems. Furthermore, most of their works only focus on reconstructing MSIs from RGBs, but ignore the process of reconstructing RGBs from MSIs. In fact, the two directions, from MSIs to RGBs and the reverse, have complementary relations. If we could take advantage of these relations, we could improve the spectrum reconstruction. In the following sections, we try to solve the above problems with our approach.

## 3. The Proposed Method

### 3.1. The Problem Analysis

Our target is to build a model to efficiently perform bidirectional translation between RGBs and MSIs. To achieve this target, we need to solve two significant challenges. One challenge is that RGBs and MSIs belong to two specific domains with different definitions and synthesis rules. There is no direct mapping relation between the two domains. The other is how to reconstruct the multispectral information, which does not exist in the RGB image. Multispectral images have more abundant information than the RGBs.

**Figure 2:**
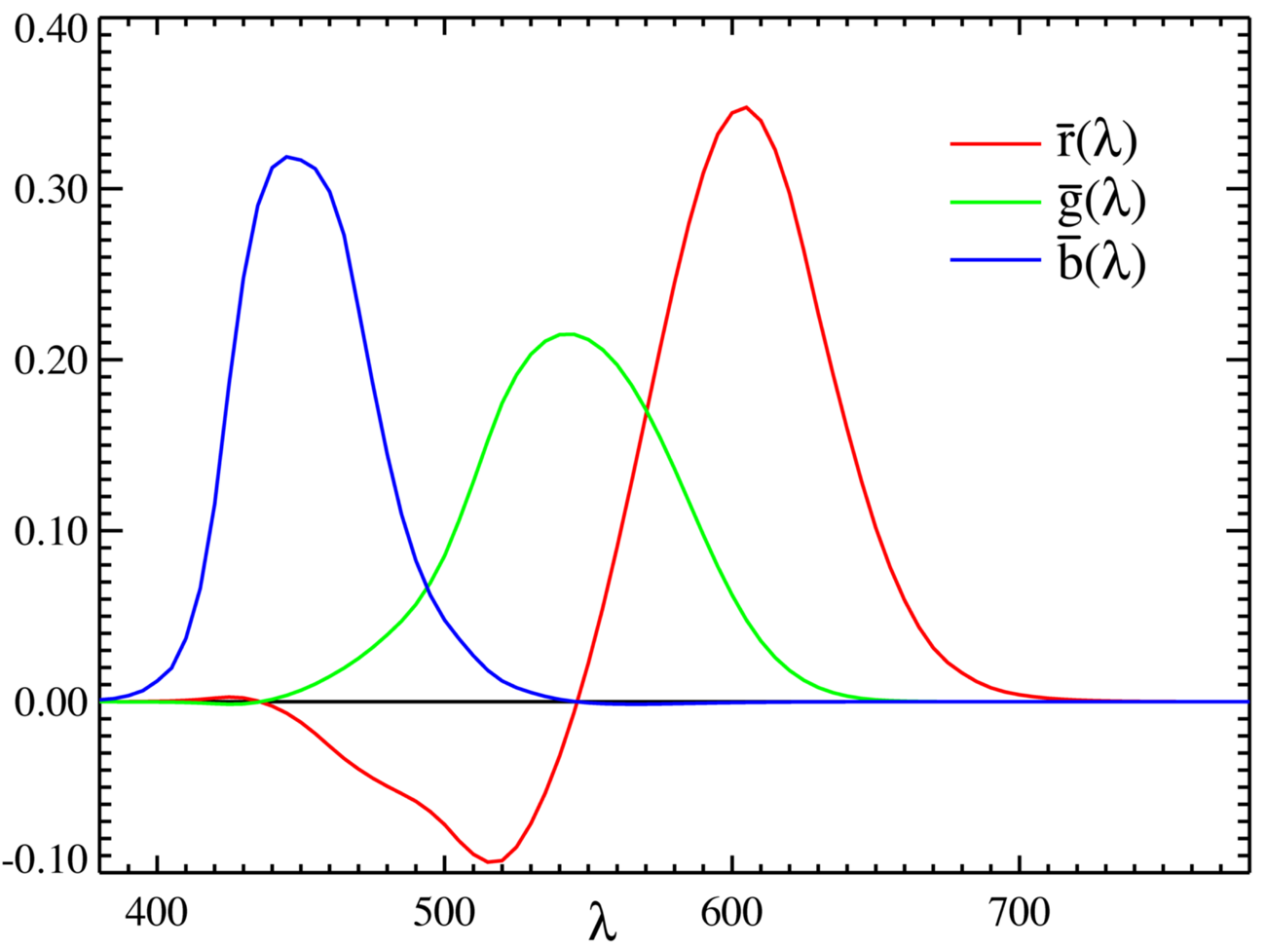
The CIE 1931 RGB color matching function [24].

Figure 2 shows the CIE 1931 RGB color matching function [24], which clearly shows the relation between the RGBs and the MSIs. Any color pixel can be created by mixing three primary colors—-red, green, and blue. On the contrary, multispectral pixels comprise different wavelength light bands. Additionally, each band only records the luminosity distribution of the specific wavelength light. Therefore, MSIs and RGBs have fundamentally different definitions and formats. There is no direct mapping relation between them.

**Figure 3:**
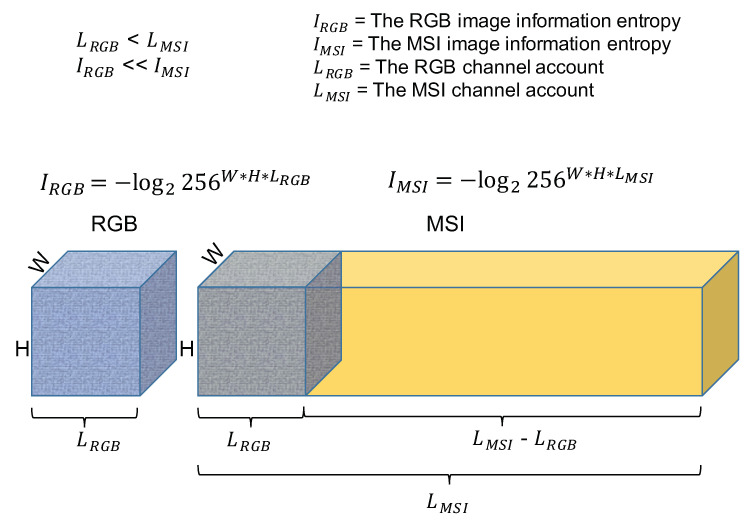
The difference of RGB and MSI image information entropies.

Additionally, Figure 3 demonstrates the second challenge. RGBs only have three bands. However, MSIs have tens or hundreds of bands. Thus, the possible space of MSIs is much larger than the possible space of RGBs. There is a massive information entropy gap between the two domains. The multispectral images contain much more information that does not exist in the RGB images. If we use RGBs to represent MSIs, we will meet the “metamerism” problem, which means one R, G, and B band combination may map to many MSI band combinations. The metamerism is an extremely underconstrained problem and will make the model’s training hard to reach convergence.

### 3.2. The Proposed Approach

**Figure 4:**
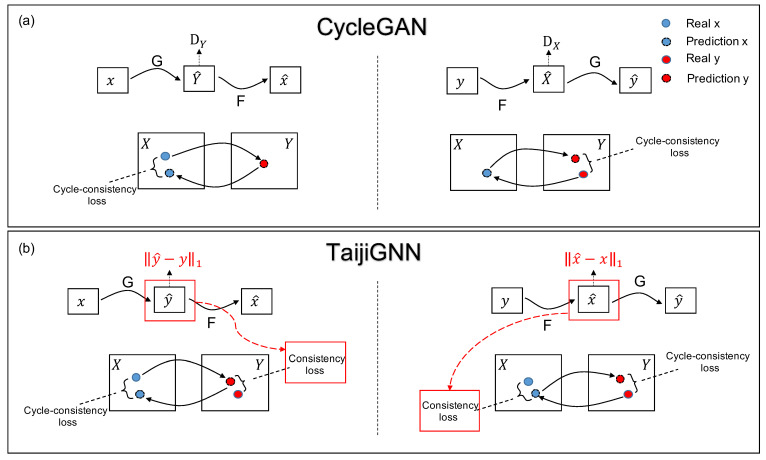
(**a**) The traditional CycleGAN’s schematic diagram [25]. (**b**) The TaijiGNN’s schematic diagram.

To solve the above two problems, we proposed TaijiGNN. Since this idea is mainly inspired by the cycle generative adversarial network (CycleGAN) and the ancient Chinese philosophy Taiji’s concept, we first introduce and compare them. Table 1 shows the symbols used in Figure 4.

Figure 4a is the original CycleGAN’s schematic diagram, which has two cycles, $x\overset{G}{\to }\hat{Y}\overset{F}{\to }\hat{x}$ and $y\overset{F}{\to }\hat{X}\overset{G}{\to }\hat{y}$. Generator *G* translates sample *x* of the domain *X* into sample $\hat{Y}$ of the domain *Y*. In the flow $x\overset{G}{\to }\hat{Y}\overset{F}{\to }\hat{x}$, we use $\hat{Y}$ instead of $\hat{y}$ since there are no paired datasets in CycleGAN; the generated sample belongs to domain *Y*, but is not specified to one and has no direct mapping relation with the original *x*. Then, we translate the $\hat{Y}$ into $\hat{x}$ with Generator *F*. Here, we aim for the $\hat{x}$ to be as close to *x* as possible, which can be used to train Generators *F* and *G*. Moreover, we use the original *Y* and the generated $\hat{Y}$ to train Discriminator $D_{Y}$ and Generator *G*. A similar occurred in the backward cycle $y\overset{F}{\to }\hat{X}\overset{G}{\to }\hat{y}$.

The most significant advantage of the original CycleGAN is that it can translate unpaired images by the cycle structure between two different domains, such as the horse and zebra [10]. However, in our case, we have paired RGBs and MSIs, which is a great advantage. Due to this advantage, we designed a novel supervised cycle-consistent generative neural network, “TaijiGNN”.

Figure 4b shows TaijiGNN’s schematic diagram, which also has two cycles, forward, $x\overset{G}{\to }\hat{y}\overset{F}{\to }\hat{x}$ and backward, $y\overset{F}{\to }\hat{x}\overset{G}{\to }\hat{y}$. The most significant difference highlighted in red shows that when we use *G* to translate the *x* into samples of domain *Y*, $\hat{y}$ replaces the $\hat{Y}$. Since paired datasets are available in this case, we can use $L_{1}$ loss to enhance the *G*’s training performance. A similar situation occurs in the backward cycle $y\overset{F}{\to }\hat{x}\overset{G}{\to }\hat{y}$. Moreover, we omit the two discriminators $D_{X}$ and $D_{Y}$, as in our case, we have the paired dataset, $L_{1}$ loss is enough to train a good model in the TaijiGNN. Extra adversary networks will bring in more noise and reduce the model’s performance.

Moreover, we anticipate that TaijiGNN will have better performance than the original CycleGAN since TaijiGNN has extra direct paired image consistency loss information, enhancing the generator’s training efficiency. Therefore, each generator of TaijiGNN can learn from the cycle-consistency loss of the source domain data and the consistency loss of the target domain data simultaneously.

Although the MSI domain and the RGB domain have different definitions and possible space sizes, its cycle architecture of TaijiGNN successfully converts comparing different domain images into comparing the same domain images. In the same domain, there are no domain matching and information supplement problems.

Furthermore, we named our neural network “Taiji” as it was mainly inspired by the ancient Chinese philosophy term “Taiji”. Taiji consists of two polarities, “Yang” (“Positive”) and “Yin” (“Negative”). Additionally, the “Yin” and “Yang” like atoms constitute every system in the universe. Within each “Yin” and “Yang” system, the “Yin” and “Yang” have opposite properties and continue to move and change all the time. The most important factor is the two polarities do not have strict boundaries and can be converted to each other under some circumstances. In the system, the “Yin” and “Yang” give rise to and complement each other to make the system reach a dynamic balance [9]. We think Taiji’s characteristics and working mechanism are consistent with our cycle-consistent generative neural network. The two generators act as Taiji’s two polarities, and during training, the two generators compose a whole system and help each other converge. Therefore, we name our neural network “TaijiGNN”.

### 3.3. The Detailed Implementation of the Approach

#### 3.3.1. The Model Architecture

**Figure 5:**
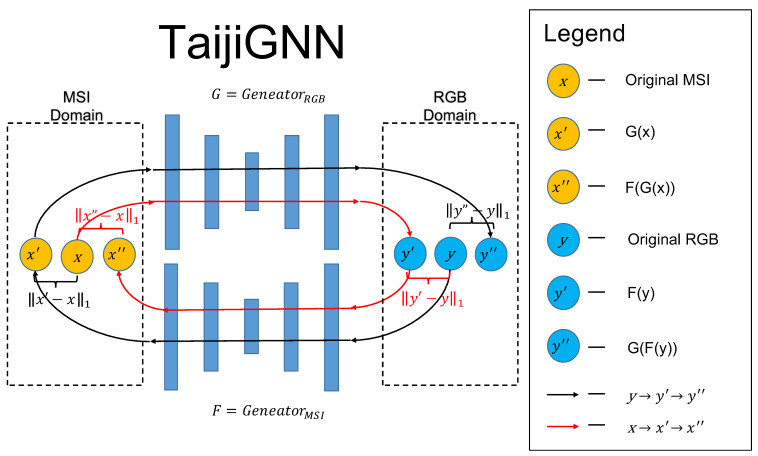
The detailed implementation of TaijiGNN.

Figure 5 is the detailed implementation of TaijiGNN. It mainly consists of two generators, *G* or $Generator_{RGB}$ (converting the MSI to the RGB) and *F* or $Generator_{MSI}$ (converting the RGB to the MSI). One generator’s output is connected with the other’s input. From different directions, they form two cycles, Cycle (3) and Cycle (4).

(3) $$\underset{\left(Original\right)}{x}\overset{G}{\to }\underset{\left(Generated\right)}{y^{\prime }}\overset{F}{\to }\underset{\left(CycleGenerated\right)}{x\prime \prime }$$

(4) $$\underset{\left(Original\right)}{y}\overset{F}{\to }\underset{\left(Generated\right)}{x^{\prime }}\overset{G}{\to }\underset{\left(CycleGenerated\right)}{y\prime \prime }$$

The significant difference between the standard CycleGAN and TaijiGNN is that TaijiGNN has two extra $L_{1}$ consistency losses, Loss $L_{RGB}$ (5) and Loss $L_{MSI}$ (6). These two losses can help to improve the two generators’ performance by calculating the mean absolute error (MAE) between the ground truth image and the generated image.

The consistency loss from MSIs to RGBs:

(5) $$L_{RGB}={∥G(x)-y∥}_{1}={∥y^{\prime }-y∥}_{1}$$

The consistency loss from RGBs to MSIs:

(6) $$L_{MSI}={∥F(y)-x∥}_{1}={∥x^{\prime }-x∥}_{1}$$

Additionally, we keep the two cycle consistency losses, Loss $L_{cycle\_MSI}$ (7) and Loss $L_{cycle\_RGB}$ (8) in our approach. Loss $L_{cycle\_MSI}$ guarantees that the cycled generated image MSI" is as close as the ground truth MSI when finishing Cycle (3). Similarly, Loss $L_{cycle\_RGB}$ ensures that the cycled generated image RGB" is as close as the ground truth RGB in Cycle (4).

The cycle consistency loss from MSIs via RGBs to MSIs:

(7) $$L_{cycle\_MSI}={∥F(G(x))-x∥}_{1}={∥F(y^{\prime })-x∥}_{1}={∥x^{\prime \prime }-x∥}_{1}$$

The cycle consistency loss from RGBs via MSIs to RGBs:

(8) $$L_{cycle\_RGB}={∥G(F(y))-y∥}_{1}={∥G(x^{\prime })-y∥}_{1}={∥y^{\prime \prime }-y∥}_{1}$$

Moreover, unlike the normal CycleGAN, TaijiGNN eliminates identity loss. In the normal CycleGAN, the input and output have identical dimensions; however, the RGB and the MSI have different dimensions in our scenario. It is neither meaningful nor possible to compare two images with different dimensions.

We also cut off two discriminator losses of the traditional CycleGAN. The reasons for this are as follows: the traditional CycleGAN does not have a paired dataset, so it must use the discriminator loss to help to train the generator. However, in our scenario, since we have a paired dataset, some L1 losses, such as MAE, have a higher performance than the discriminator loss. Additionally, training through discriminator losses is an indirect training method, which will make the generated image blurry. Overall, direct training is better than indirect training.

The two generators of TaijiGNN take the core roles of converting images between the MSI domain and the RGB domain. The choice of architecture directly determines the performance of the system. We tried several mainstream architectures, including the convolutional neural netwok (CNN), residual neural network, U-Net and Multi-Layer Perception (MLP). The MLP has the best performance.

Regarding the above phenomenon, our explanation is as follows: In our scenario, the main target is to produce the spectral super-resolution, not the spatial super-resolution. The traditional neural networks based on CNN can effectively extract information distributed on the two spatial dimensions, such as shape. However, the required spectral information is distributed on the third dimension. The neural networks based on 1-D MLP focus on extracting spectral features, neglecting the 2-D spatial information. Therefore, 1-D MLPs often obtain better results than 2-D CNNs.

Based on the above analysis and our real verifications, TaijiGNN’s two generators adopt MLP architecture. The detailed network architectures are shown in Table 2 and Table 3, respectively.

#### 3.3.2. The Model Training

The core training goal is to make the two generators, $G_{RGB}$ and $F_{MSI}$, converge. The primary approach is to apply gradient descent on the previous four losses, $L_{RGB}$ (5), $L_{MSI}$ (6), $L_{cycle\_MSI}$ (7), and $L_{cycle\_RGB}$ (8).

**Figure 6:**
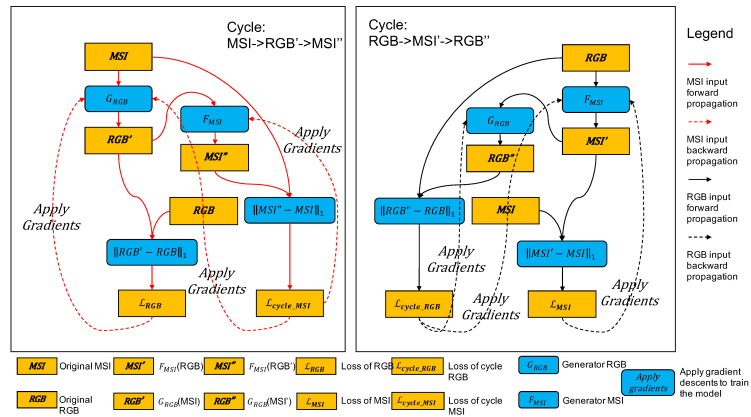
The detailed training flow of TaijiGNN.

Figure 6 demonstrates the whole training flow of TaijiGNN. From Figure 6, we can find that the whole training flow consists of two symmetrical cycles, Cycle $x\left(MSI\right)\overset{G}{\to }y^{\prime }\left(RGB^{\prime }\right)\overset{F}{\to }x^{\prime \prime }\left(MSI^{\prime \prime }\right)$ marked with red solid and dash lines, and Cycle $y\left(RGB\right)\overset{F}{\to }x^{\prime }\left(MSI^{\prime }\right)\overset{G}{\to }y^{\prime \prime }\left(RGB^{\prime \prime }\right)$ highlighted with black solid and dash lines. Since they are symmetrical, we only describe Cycle $y\left(RGB\right)\overset{F}{\to }x^{\prime }\left(MSI^{\prime }\right)\overset{G}{\to }y^{\prime \prime }\left(RGB^{\prime \prime }\right)$ in detail.

Cycle $y\left(RGB\right)\overset{F}{\to }x^{\prime }\left(MSI^{\prime }\right)\overset{G}{\to }y^{\prime \prime }\left(RGB^{\prime \prime }\right)$ starts from inputting an RGB image into Generator $F_{MSI}$, which obtains a reconstructed MSI image $MSI^{\prime }$. After this operation, the flow splits into two branches. One branch calculates $L_{MSI}$ by computing the MAE between the original MSI and the generated $MSI^{\prime }$. Additionally, when we obtain Loss $L_{MSI}$, we can apply gradient descents to update Generator $F_{MSI}$. The other branch inputs the $MSI^{\prime }$ into Generator $G_{RGB}$ to obtain the cycled generated $RGB^{\prime \prime }$ and then to calculate Loss $L_{cycle\_RGB}$ by comparing the original RGB with the cycled generated $RGB^{\prime \prime }$. Once we obtain Loss $L_{cycle\_RGB}$, we can apply gradient descents to update Generator $F_{MSI}$, and Generator $G_{RGB}$. A similar training flow occurs in Cycle $y\left(RGB\right)\overset{F}{\to }x^{\prime }\left(MSI^{\prime }\right)\overset{G}{\to }y^{\prime \prime }\left(RGB^{\prime \prime }\right)$.

Figure 6 demonstrates a classical TaijiGNN training epoch. In each training epoch, Generator $G_{RGB}$ and Generator $F_{MSI}$ are updated three times, respectively. In detail, Generator $G_{RGB}$ is updated by applying gradients, Loss $L_{RGB}$, Loss $L_{cycle\_MSI}$ and Loss $L_{cycle\_RGB}$, respectively. Similarly, Generator $F_{MSI}$ is updated by applying gradients, Loss $L_{MSI}$, Loss $L_{cycle\_RGB}$ and Loss $L_{cycle\_MSI}$. Therefore, after several epochs of training, Generator $G_{RGB}$ and Generator $F_{MSI}$ eventually converge.

## 4. Experiments

The CAVE [26] and ICVL [15] are the two most classical spectral reconstruction experiment datasets. To compare our work with state-of-the-art works equally and evaluate our approach’s performance thoroughly, we also selected CAVE and ICVL as our experiment datasets. We demonstrate the detailed experiment process and outcomes in the coming subsections.

### 4.1. The Experiment Requisites

We list all the hardware devices and software involved in Table 4 and Table 5, respectively.

Since the Graphics Processing Unit (GPU) plays a vital role in the training and the inferencing, we provide a detailed introduction. Our GPU is Nvidia TITAN Xp, which has 3840 parallel computing cores with a 1582 MHz frequency in our experiment. The GPU memory has 12 GB GDDR5, 11.4 Gbps speed, and 547.7 GB/s bandwidth. In this experiment, since the image processing contains many high-density computing jobs, we leveraged the GPU to accelerate the training and inferencing process. Moreover, in Appendix A, we provide a summary of TaijiGNN’s performance on the GPU and the CPU.

### 4.2. The CAVE Dataset

#### 4.2.1. The Dataset Processing and the Hyperparameters Setting

The CAVE dataset was created by Columbia University Computer Vision Laboratory. It consists of 32 scenes taken indoor, and the scenes are divided into five sections: real and fake, stuff, paints, skin and hair, and food and drinks. Each scene is constituted of a multispectral image with 31 bands and an RGB image with 3 bands. The MSI’s 31 bands span from 400 nm to 700 nm visible light at 10 nm intervals. Furthermore, each image’s spatial resolution is 512×512 pixels [26].

**Figure 7:**
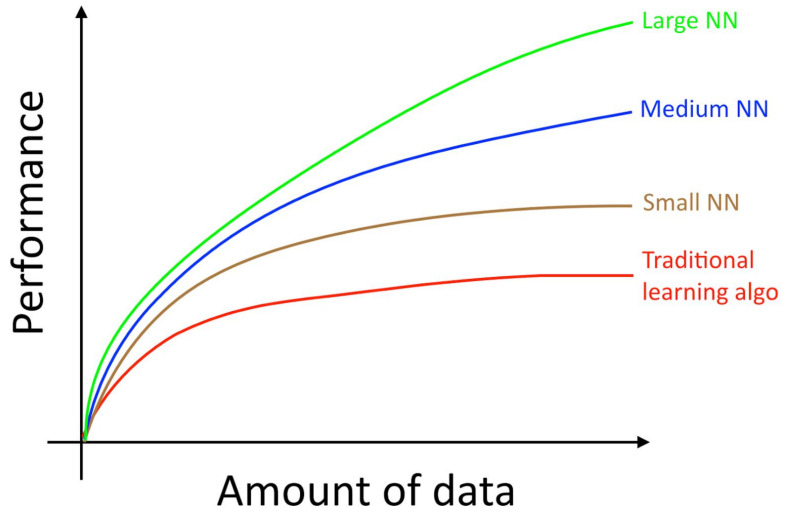
The relevance between the amount of data and the model’s performance [27].

For easy processing and comparison, we first converted the original PNG image dataset into the NumPy array, which contains 32 rows (scenes), 512×512 (spatial resolution), and 34 columns (bands or channels). The 0–30 columns match the 400 nm, 410 nm, …, 700 nm MSI channels. Simultaneously, the 31–33 columns map to the Red, Green, and Blue channels. Moreover, according to the scene’s row number, we separated the dataset into two equal-sized parts. Images with even indexes, 0, 2, 4, …, 30 were placed into one group, whereas images, 1, 3, 5, …, 31, with odd indexes, were placed into another group. We rotated to use the odd group and the even group to train and test the model. Generally speaking, the amount of training data is proportional to the performance of the model. Figure 7 demonstrates this phenomenon [27]. To achieve better performance, the training and testing ratio is often set as 80%:20% or 70%:30%. However, increasing the ratio of the training data unavoidably leads to overfitting results and restricts application domains.

We chose a more challenging method to split the CAVE, half for training, half for testing, which is the same as in the work of Kin et al. [21]. Moreover, Kin et al.’s conditional GAN is similar to our TaijiGNN. Both have generative network architecture. Therefore, their work was selected as the primary baseline. Furthermore, Arad et al. created the ICVL dataset and declared that they achieved advantage outcomes at that time [15]. Berk et al. announced that they were the first to successfully reconstruct a multispectral image from an RGB image taken in unrestricted configuration [20]. Therefore, Arad and Berk’s works were also selected for comparison.

Moreover, we performed qualitative and quantitative evaluations of the bi-directional conversion between the RGB image and the multispectral image in the experiment.

Additionally, the detailed training parameter settings were as follows: There were 300 training epochs, and the training and the inferencing batch size was 10,240. We adopted the Adam optimizer, whose learning rate is 2× 10${}^{-4}$.

#### 4.2.2. The Qualitative Evaluation

The qualitative evaluation was conducted in two stages: reconstructing multispectral images from RGB images and the reverse. Compared with related work introduced in Section 2, one advantage of TaijiGNN is that it can finish the above two spectral translations simultaneously.

**Figure 8:**
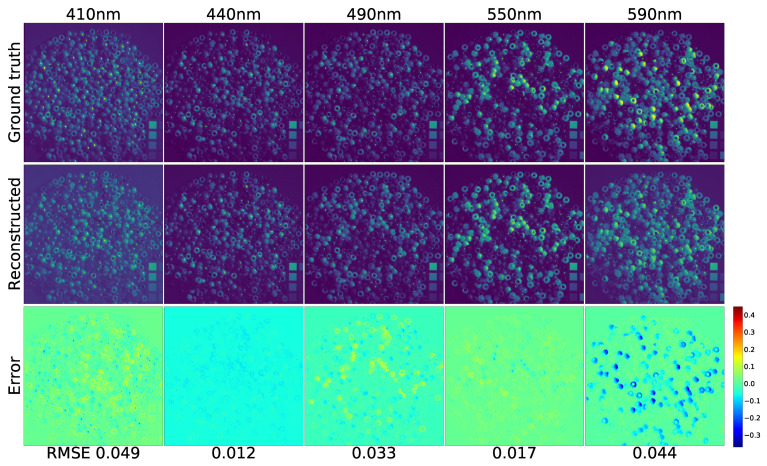
The effects of reconstructing the 5 chosen spectrum channels from an RGB image. (The original input RGB is shown in the top-left image of Figure 9).

**Figure 9:**
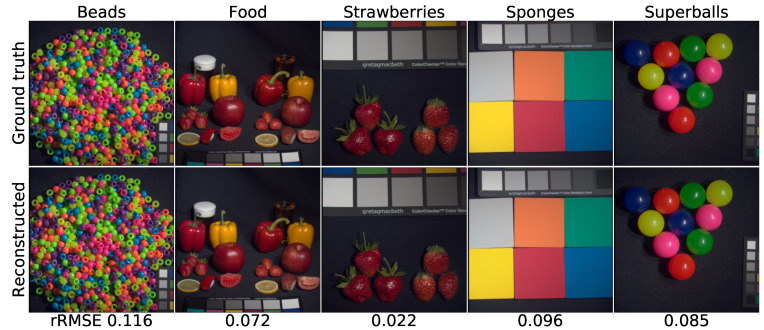
The effects of reconstructing the 5 chosen RGB images from the multispectral images.

**Figure 10:**
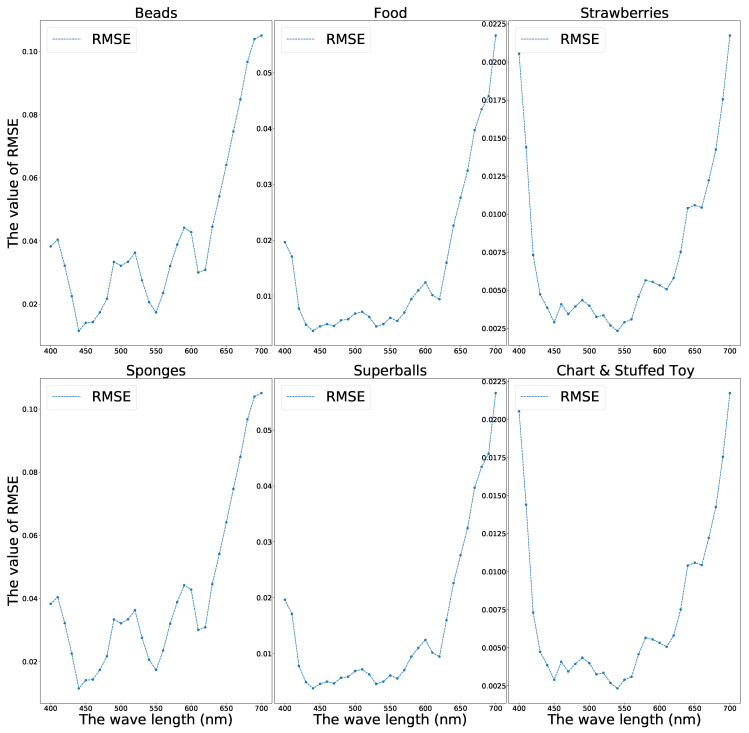
The RMSE wavelines of multispectral image restoration with the CAVE database.

Figure 8 is the qualitative effect of reconstructing the MSI from the RGB with Generator $F_{MSI}$. As in [21], five spectral bands of image “beads” were chosen as samples. Additionally, the ground truth input RGB image was shown in the top-left image of Figure 9. The five selected bands span from 400 nm to 700 nm with approximate equal intervals. Since the naked eye cannot identify lights with such subtly different wavelengths, it becomes a prevalent and standard method to select several bands to present the qualitative spectral reconstruction results [15,20]. Moreover, in Section 4.2.3, we calculated the RMSE for the whole spectrum, from 400 nm to 700 nm, and drew the RMSE curve in Figure 10.

Additionally, we use the error map to present the degree of dissimilarity between the ground truth image and the reconstruction image. The error maps were generated by subtracting the original image from the forecast image. Additionally, we set the colormap equaling “jet” to pseudocolor the error images. The blue represents the positive error, the green signifies the zero error, and the red denotes the negative error. Furthermore, we noticed that our MSI reconstruction images are clearer than those on the five selected bands compared to the same qualitative experiment conducted by Kin et al.

Moreover, with Generator $G_{RGB}$, we can quickly reconstruct RGBs from MSIs. Five of the same images, “Beads”, “Food”, “Strawberries”, “Sponges”, and “Superballs” were selected to evaluate the reconstruction quality of $G_{RGB}$ in Figure 9. Since we cannot evaluate our result and that of Kin et al. by naked eye, we had to calculate every scene’s relative RMSE and list them under the figure’s bottom line. The result is better than that of Kin et al. [21].

#### 4.2.3. The Quantitative Measurement

After a full investigation on the measurements of related research, we decided to adopt the Structural Similarity Index (SSIM), Peak Signal-to-Noise Ratio (PSNR), and Root Mean Square Error (RMSE) as the main quantitative metrics.

The dataset processing has been explained previously. We rotated odd position samples and even position samples to train and test the model. Additionally, the training and testing were performed three times and the average value was calculated. We conducted the experiments in two directions, RGB to MSI and MSI to RGB and compared our results with the following three state-of-the-art works: Berk et al. [20], Kin et al. [21], and Arad et al. [15]. The final results are listed in Table 6 and Table 7, respectively.

From Table 6, a phenomenon is noticeable, the results of Arad et al. ranked first and our results ranked second are very close. Our explanations are as follows:

First, Arad et al. did not clearly describe how they split the CAVE dataset for training and evaluation. According to our speculation, their splitting ratio should be 70–80% for training and 20–30% for testing. As shown in Figure 7, model training with more data usually yields a higher performance. Therefore, their splitting ratio gives their model more advantages.

Second, before training, the method of Arad et al. must first take a hyperspectral prior sampled from each image. The CAVE dataset’s sampling ratio is 3.8% for every image, which is equivalent to knowing the answers to some questions before testing. This kind of method would be helpful to highly improve their model’s effectiveness. However, it would easily lead to overfitting, causing it to be useless in reconstructing images in the unknown dataset.

On the contrary, Kin et al. adopted the same splitting dataset ratio as us, which was half for testing and half for training, and isolated the testing data and the training data strictly, which indicated that the model during training would not receive any information from the testing data. Therefore, TaijiGNN would have better performance in the unseen dataset. Therefore, it is not accurate to conclude that the approach of Arad et al. is superior to ours.

Moreover, Kin et al. adopted the same dataset splitting ratio as us. Compared with their RMSE result, TaijiGNN was reduced 30% in converting the RGB image to the multispectral image and reduced 69% in the reverse direction.

Furthermore, identical to Kin et al., we selected six different scenes, “Food”, “Beads”, “Sponges”, “Strawberries”, “chart & Stuffed Toy”, and “Superballs”, to investigate the reconstruction performance changing with the scene and the wavelength. Figure 10 demonstrates the RMSE changes in the six scenes.

From Figure 10, we can find that the model reconstruction performances have some differences in various scenes. However, the shapes of the RMSE curves are similar. Generally, the generator performs better when reconstructing the band images whose wavelengths are 450 nm–650 nm. We provide the following reasons for this: From the CIE 1931 color matching function (Figure 2), we may notice that most red, green, and blue components are distributed mainly in the wavelength range of 450 nm–650 nm and allocate scarce elements to the two ends of the spectrum. One pixel of RGB image comprises three primitive colors, red, green, and blue, containing limited information about the two ends of the spectrum that may cause bad reconstructing behavior near the two-end spectral zone.

### 4.3. The ICVL Dataset

#### 4.3.1. The Dataset Processing and the Hyperparameters Setting

The ICVL dataset was created by the interdisciplinary Computational Vision Lab of Ben-Gurion University. Its latest version consists of 201 scenes, most of which were taken outdoors by a Kappa DX4 hyperspectral camera. Additionally, the dimensions of every multispectral image are 1392 pixel height, 1300 pixel width, and 519 band depth. The 519 bands are distributed from 400 nm to 1000 nm in approximately 1.25 nm increments [15].

In this experiment, we adopted a reduced version of the ICVL dataset, supplied by the authors [15]. The 519 bands were reduced to the 31 bands with approximately 10 nm intervals from 400 nm to 700 nm, which has two benefits: decreasing the computation cost and comparing them to former baselines easily [15]. Moreover, we used the CIE 1964 color matching function to generate the RGB images from the 519 band MSIs and the 31 band MSIs. The difference between them is too small to identify. The RMSE of the two kinds of generated RGB images is approximately 0.00014.

First, we randomized the order of the 201 scenes and divided them into six groups. Each of the first five groups, 1st, 2nd, 3rd, 4th, and 5th, had 32 samples. However, the 6th group contains 41 samples. We iterated one of the 1st–5th groups to train the model and the remaining four groups plus the 6th group to test the model. For instance, when taking the 3rd group to train the model, the 1st, 2nd, 4th, 5th, and 6th groups were used to test the model. This kind of dataset processing aims to make the evaluation thorough and non-overlapping. Meanwhile, the splitting ratio of training and testing for the dataset reached 32:169, which means thirty-two samples to train the model and one-hundred-and-sixty-nine samples to test the model.

Additionally, as in Section 4.2.1, every 32 image training group was further separated into two equal-size parts. Images with even indexes, 0,2,4,…,30 were placed into one group, whereas images with odd indexes, 1,3,5,…,31 were placed into another group. We rotated the odd group and the even group to train and test the model.

Additionally, a further challenge was made to decrease the spatial resolution of training samples from [1392 (height), 1300 (width)] to [512 (height), 512 (width)]. To achieve the above target, we separate the original sample ([1392,1390]) into four equally sized ([512, 512]) blocks, top-right, top-left, bottom-right, and bottom-left. For each time training, we randomly selected one of the four blocks. In our experiment, we performed the training four times to ensure all four blocks would be chosen. The final training group’s dimensions became [32 (sample), 512 (height), 512 (width), 34 (MSI+RGB bands)]. However, to compare the state-of-the-art works equally, the testing group’s dimensions were still [169,1392,1300,34]. Therefore, the ratio of the training samples and the testing samples achieved 32:169. As far as we know, until now, no one adopted such a small splitting ratio of training and testing in the ICVL dataset.

Furthermore, to completely assess the approach, bi-directional conversions between the multispectral image and the RGB image were performed. Additionally, qualitative and quantitative assessments were conducted simultaneously. Additionally, the works of Zhiwei et al. [17], Arad et al.’s [15], and Berk et al.’s [20] were selected as references. Moreover, Zhiwei et al. declared that their HSCNN’s outcomes surpassed those of other works [17].

Similarly, the detailed training parameter settings were as follows: the training epochs were 300, and the training and inferencing batch sizes were both 10,240. We adopted the Adam optimizer, whose learning rate is 2× 10${}^{-4}$.

#### 4.3.2. The Qualitative Evaluation

The ICVL dataset reconstruction qualitative evaluation consists of two stages: reconstructing the MSIs from the RGBs and the reverse. Before the experiment, the training data and the testing data were effectively processed according to Section 4.3.1.

**Figure 11:**
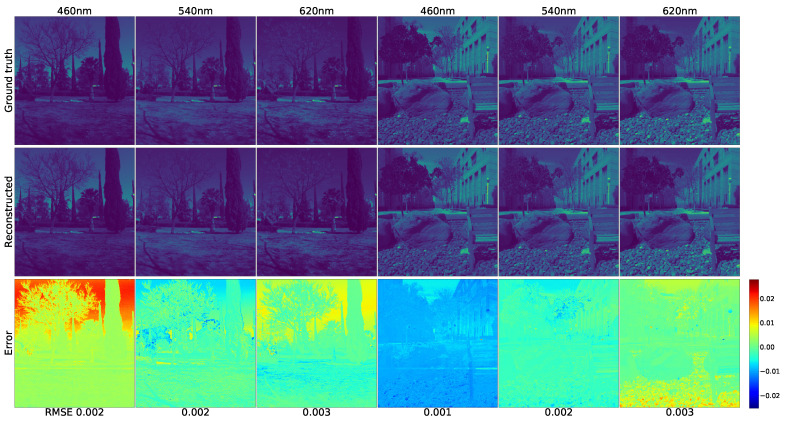
The effects of reconstructing the 3 chosen ICVL spectrum bands from the 2 scenes. (The two original input RGB images are shown in the top-left 2 images of Figure 12.)

**Figure 12:**
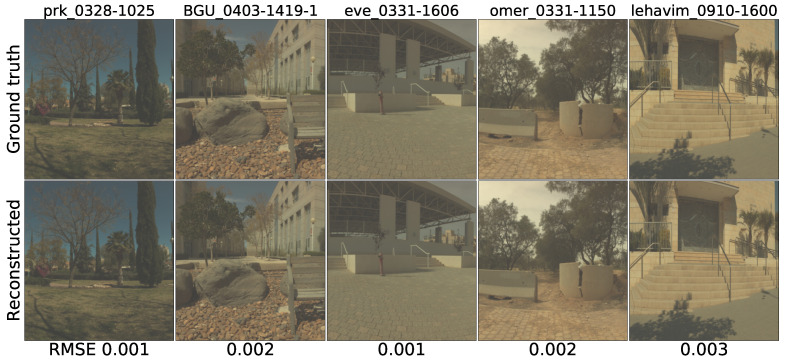
The effects of reconstructing the 5 chosen ICVL RGB images from the multispectral images.

As the ICVL database was created by Arad et al., who claimed that their approach obtained superior results [15]. Therefore, the work of Arad et al. was chosen to be the main baseline. Figure 11 was created with Generator $F_{MSI}$ according to the format of Figure 4 in the work of Arad et al [15]. Scene “BGU_0403-1419-1” and scene “prk_0328-1025” were selected as the samples. Moreover, 620 nm, 540 nm, and 460 nm bands were selected to exhibit the qualitative restoration performance. The top-left 1st RGB-image “prk_0328-1025” and 2nd RGB-image “BGU_0403-1419-1” in Figure 12 are the two original input RGBs.

The error maps were generated by subtracting the original image from the forecast image. Additionally, we set the colormap “jet” mode to pseudocolor the error images. The blue represents the positive error, the green signifies the zero error, and the red denotes the negative error.

Furthermore, when we compare the two ICVL figures [11,12] with the two previous CAVE figures [8,9], respectively, we may notice that the identical method performs more effectively on the ICVL scenes than on the CAVE scenes. We think that the main reason for this is that since most of the CAVE images are taken indoors, only a few bright objects contain useful information, and the main dark background contains scarce data to train the model. On the contrary, most of the ICVL images were captured outdoors. In addition to the objects, the background also includes a large amount of useful information that would help to improve the model training performance.

#### 4.3.3. The Quantitative Measurement

Similar to the evaluation of the CAVE model, we picked four standard quantitative measurements, RMSE, PSNR, SSIM, and added relative RMSE (nRMSE or rRMSE) to evaluate the ICVL model’s performance.

Moreover, we prepared two experiments and several comparisons with the leading related works to show TaijiGNN’s advantages.

We compared the results of TaijiGNN with those of the two top works, the HSCNN by Zhiwei et al. and the sparse coding by Arad et al., during the first experiment. To make the comparison impartial, we adopted an equal or higher level to process the ICVL dataset, which is described in the following paragraphs.

Arad et al. performed all their experiments on a dataset named “complete set”, owning 102 scenes. After careful comparison, we noticed that in their supplied dataset, scene “lst_0408-0924” lost [15]. Therefore, we removed it from the dataset. The final scene amount of the dataset became one-hundred-and-one. Additionally, they selected 59 scenes from the dataset and assigned them to 5 domains: Indoor, Park, Rural, Urban, and Plant-life. Their experiment strategy is domain-specific, selecting a scene from a domain for testing, and the remaining scenes of the domain were used to train the model. Additionally, the above steps were repeated until all scenes of that domain had been picked. Finally, Arad et al. measured the model’s performance by the average relative RMSE (rRMSE). According to their claim, the model trained by the domain-specific subsets has better results than the complete dataset model [15].

Zhiwei et al. processed the ICVL dataset more generally when evaluating their approach, “HSCNN”. Their dataset contains 200 images, including the complete set of previous images. Their experiment has two phases: In the first phase, they trained the model with 141 images and tested on the 59 domain-specific images. In the second phase, they trained the model with the 159 images, excluding the non-domain-specific images in the complete set, and tested the obtained model on the remaining 41 images. In this way, the training images and the testing images were precisely isolated, and the domain-specific restriction was eliminated [17].

We extended the dataset splitting method of Zhiwe et al. First, we updated the total dataset to 201 images. Second, we reduced the training image number to 100 images and assessed the neural network on the complete set (101 scenes) of Arad et al., containing the fifty-nine domain-specific scenes. The training and testing images were rigorously isolated. Third, in each evaluation, we randomly sampled 32 images from the 100 training images and decreased their spatial size from [1392 (height), 1390 (width)] to [512 (height), 512 (width)]. The detailed process is as follows: First, we separate the original image ([1392, 1390]) into four equally sized ([512, 512]) frames, top-right, top-left, bottom-right, and bottom-left, to achieve the above-mentioned spatial size translation. For each time training, we randomly selected one of the four blocks. Although the training image spatial resolution was 512×512, the test images maintained the 1392×1300 spatial resolution, which is one of our approach’s advantages. To thoroughly cover all the images, we performed the evaluation 5 times and calculated their average values.

Table 8 summarizes the above three approaches’ results of reconstructing MSIs from RGBs. Our approach, TaijiGNN, surpasses the other two in most subsets and the complete set. Furthermore, our training and testing ratio is the lowest.

We increased the challenge in the second experiment to fully explore TaijiGNN’s potential. We removed the first experiment dataset splitting method and adopted the dataset splitting method in Section 4.3.1.

We randomized the order of the 201 scene samples and divided them into six groups. Each of the first five groups, 1st, 2nd, 3rd, 4th, and 5th, had thirty-two samples. However, the 6th group contained forty-one samples. We iterated one of the 1st–5th groups to train the model and the remaining four groups with the 6th group to test the model. For instance, when taking the 3rd group to train the model, the 1st, 2nd, 4th, 5th and the 6th groups were used to test the model. This dataset processing aims to make the evaluation thorough and non-overlapping. Meanwhile, the splitting ratio of training and testing for the dataset reached 32:169, which means thirty-two samples to train the model and one-hundred-and-sixty-nine samples to test the model.

Additionally, every 32-image training group was further separated into two equally sized parts. Images with even indexes, 0,2,4,…,30 were placed into one group, whereas images with odd indexes, 1,3,5,…,31 were placed into another group. We rotated the odd group and the even group to train and test the model.

Additionally, we decreased the spatial resolution of the training samples from [1392 (height), 1300 (width)] to [512 (height), 512 (width)] to increase the training speed, which unavoidably increased the convergence difficulty. A detailed description of the dataset processing is presented in the previous paragraphs. The final training group dimension bacame [32,512,512,34]. However, the testing group dimension maintained [169,1392,1300,34]. Therefore, the splitting ratio of the training group and the testing group achieved 32:169. To make the evaluation cover the whole dataset, we conducted four experiments on every group and calculated their average values.

We present the final bi-directional conversion results between MSIs and RGBs in Table 9. To the best of our knowledge, such a training and testing experiment with such a low splitting ratio has not been conducted to date. Compared with the CAVE dataset outcomes (Table 6 and Table 7), we can immediately discover that the performance of TaijiGNN is much higher in the ICVL database than in the CAVE database, which affirms the previous analysis in Section 4.3.2.

**Figure 13:**
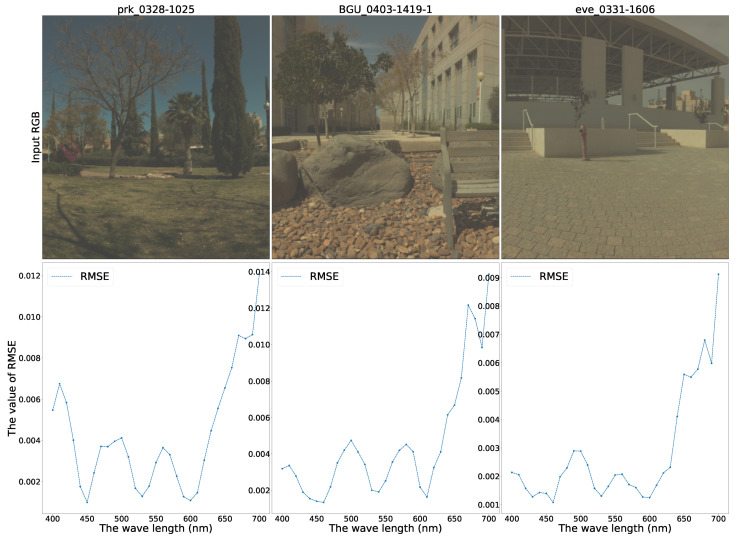
The RMSE waveline of multispectral image restoration on the ICVL database.

Moreover, to investigate the rule of RMSE changing with the scenes and the light wavelength, image “prk_0328-1025”, image “BGU_0403-1419-1”, and image “eve_0331-1606” were chosen to assess the 31 band multispectral restoration RMSEs, respectively, and the corresponding RMSE curves were drawn in Figure 13. Additionally, Arad et al. adopted the image “prk_0328-1025” and image “BGU_0403-1419-1” in their paper [15] and image “eve_0331-1606” was adopted in the work of Zhiwei et al. as an example [17].

Observing Figure 13, we can notice that the model’s reconstruction performance performs poorly within the long wavelength spectrum. Our explanation is as follows: Since the three images were taken outdoor in daylight, the backgrounds and environments may generate large amounts of thermal noise and long-wave light, disturbing the collection of useful long-wave light from objects. When we recorded the long-wavelength light in the daytime, we simultaneously saved a large amount of background noise, mainly from the sunlight and the background. Too much background noise cuts down the MSI reconstructing performance.

## 5. Limitations

TaijiGNN has four losses, $L_{RGB}$ (5), $L_{MSI}$ (6), $L_{cycle\_MSI}$ (7), and $L_{cycle\_RGB}$ (8). We took advantages of these losses to train Generator $G_{RGB}$ and Generator $F_{MSI}$. In the experiment section, we only set a 1:1:1:1 ratio for the four losses. However, we are not confident that the 1:1:1:1 ratio is the best, as we did not fully understand their roles during the training process.

To make clear the roles of the four losses, we maintained the values of $L_{cycle\_MSI}$ (7) and $L_{cycle\_RGB}$ (8), and multiplied parameter β by $L_{MSI}$ (6) and $L_{RGB}$ (5). In this way, we obtained the total loss formula (9). Moreover, we tuned the β value to observe the RMSE changing against the β.

(9) $$L_{Totally}=\beta \times \left(L_{MSI}+L_{RGB}\right)+L_{cycle\_MSI}+L_{cycle\_RGB}$$

We performed two experiments on the CAVE dataset. One is reconstructing MSIs from RGBs, and the other is reconstructing RGBs from MSIs. The results are listed in Table 10 and Table 11, respectively.

**Figure 14:**
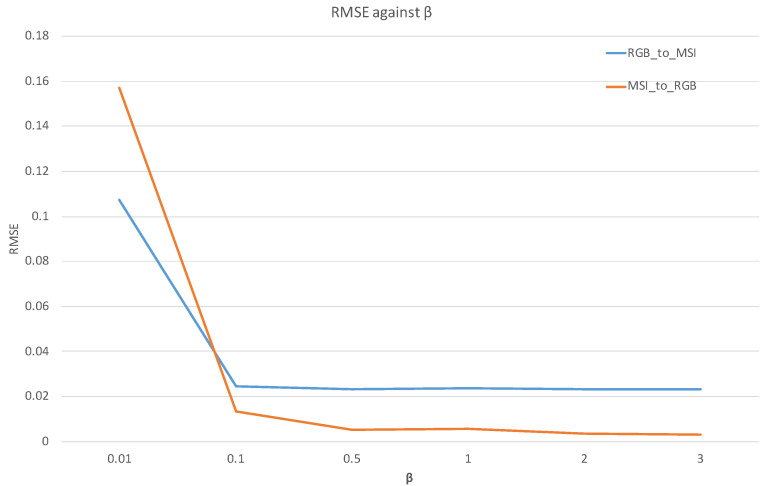
The RMSE against the β on the CAVE dataset.

Moreover, the two tables’ contents are shown in lines in Figure 14. From Figure 14, we can quickly conclude that loss $L_{MSI}$ (6) and loss $L_{RGB}$ (5) play larger roles in the training process. When we decreased their percentages, the performances of the two generators decreased quickly.

Moreover, since this is our first time investigating the feasibility of using the cycled neural network to translate between MSIs and RGBs, we adopted a symmetrical architecture for simplicity. Generator $G_{RGB}$ and Generator $F_{MSI}$ adopt the same neural network structures. However, we know that reconstructing MSIs from RGBs is much more complex than reconstructing RGBs from MSIs. It is an asymmetrical problem. The symmetrical structure may restrict the performance of the neural network. We will investigate asymmetrical neural network architecture to improve the performance of TaijiGNN in the future.

Furthermore, if the spectral range is above 700 nm, it can be reconstructed by RGB. However, it needs corresponding multispectral images, which are taken above 700 nm. Currently, both the CAVE dataset and the ICVL dataset are taken within 400 nm–700 nm. Since both the datasets do not have any information above 700 nm, it is impossible to reconstruct the multispectral images above 700 nm from the RGB of the CAVE dataset and the ICVL dataset. The CAVE and the ICVL datasets are the most popular datasets to verify spectral super-resolution performance. Since it is not easy to find a suitable dataset above 700 nm, but few related works have done it, it is hard to find a baseline to compare and verify our approach. We plan to extend this study and evaluate our work above 700 nm and below 400 nm in the future.

## 6. Conclusions and Future Work

In this paper, we first introduced the two challenges in the bi-directional conversion between MSIs and RGBs. One challenge is that MSIs and RGBs belong to two different domains and have different rigid definitions, and direct translation is difficult to accomplish. The other is that reconstructing MSIs from RGBs is a severely underconstrained process due to the colossal information entropy gap.

To address the two above-mentioned challenges, we proposed a new approach, “TaijiGNN”, which can convert the problem of comparing different domain images into comparing the same domain images by the cycle neural network architecture. In this way, the two above-mentioned problems can be solved naturally. In addition, we used a well-designed multilayer perceptron neural network to substitute the convolutional neural network when implementing the generators to make them simpler and more efficient. Besides, we cut off the two traditional CycleGAN identity losses to fit the spectral image translation and added two consistency losses to enhance the model training.

Finally, several experiments were conducted on the two classical datasets, CAVE and ICVL, to evaluate our method thoroughly. Under the same validation configuration as the previous state-of-the-art, much less training data enable our approach to obtain similar outcomes in the CAVE dataset and gain dramatic improvements in the ICVL dataset.

In the future, we plan to extend our work in the following directions: First, we will investigate asymmetrical neural network architecture to improve the performance of TaijiGNN and the impacts of the hyperparameter change. Second, we will extend our approach to the spectral image dataset above 700 nm and below 400 nm. Third, we will use GPU and FPGA to accelerate the training and inferencing processes and investigate the performance improvements.

## Figures and Tables

**Table 1:** The symbols table of Figure 4.

Symbol	Meaning
*x*	Original sample from *X* domain
*y*	Original sample from *Y* domain
$\hat{x}$	Sample generated by Generator *F* and paired with *y*
$\hat{y}$	Sample generated by Generator *G* and paired with *x*
*X*	Domain *X*
*Y*	Domain *Y*
$\hat{X}$	Sample generated by Generator *F* and unpaired with *y*
$\hat{Y}$	Sample generated by Generator *G* and unpaired with *x*
*G*	Generator, translate samples from domain *X* into domain *Y*
*F*	Generator, translate samples from domain *Y* into domain *X*
$D_{X}$	Discriminator of domain *X*
$D_{Y}$	Discriminator of domain *Y*

**Table 2:** The architecture of Generator $F_{MSI}$.

	Layer	Input	Output	Activation Function
	Dense	RGB(3)	512	Leaky Relu
**$F_{MSI}$**	Dense	512	512	Leaky Relu
	Dense	512	MSI(31)	tanh

**Table 3:** The architecture of Generator $G_{RGB}$.

	Layer	Input	Output	Activation Function
	Dense	MSI(31)	512	Leaky Relu
**$G_{RGB}$**	Dense	512	512	Leaky Relu
	Dense	512	RGB(3)	tanh

**Table 4:** The hardware list.

Hardware	Models
Central Processing Unit	Xeon(Intel) E5-1620 (3.5 GHz)
Graphics Processing Unit	TITAN-Xp (NVIDIA)
RAM	DDR4 32 GB (Samsung)
Mechanical Hard Disk Drive	Westwood 4TB
SSD	512 GB (Intel)

**Table 5:** The software list.

Software	Version
Operating system	Ubuntu 18.04.5 LTS
Programming language	Python 3.6.9
Machine learning framework	Tensorflow with GPU 2.3.1
GPU programming framework	Nvidia CUDA 11.2.0

**Table 6:** The quantitative measurement of converting RGB image to multispectral image [15,20,21].

	Berk	Kin	Arad	Ours
Method	CNNs	cGANs	Sparse coding	TaijiGNN
Percentage of training and testing	-	50%:50%	-	**50%:50%**
RMSE∼(0–255)	-	8.0622	**5.4**	5.656
RMSE∼(0–1)	0.038	-	-	**0.022**
SSIM	0.94	-	-	**0.975**
PSNR	28.78	-	-	**33.91**

**Table 7:** The quantitative measurement of converting multispectral image to RGB image [20,21].

	Berk	Kin	Ours
Method	CNNs	cGANs	TaijiGNN
Percentage of training and testing	-	50%:50%	**50%:50%**
RMSE∼(0–255)	2.55	5.649	**1.727**
RMSE∼(0–1)	0.038	-	**0.0068**
SSIM	0.94	-	**0.99**
PSNR	28.78	-	**45.07**

**Table 8:** The three approach results of reconstructing MSIs from RGBs on the ICVL dataset.

**Author**	Arad et al. [15]	Zhiwe et al. [17]	Ours
**Methods**	Sparse coding	HSCNN	TaijiGNN
**Percentage of training and testing**	-	141:59	**32(train):68(validation):101(test)**
**Evaluation metric**	rRMSE	rRMSE	rRMSE
**Park subset**	0.0589	0.0371	**0.0347**
**Urban subset**	0.0617	0.0388	**0.0335**
**Indoor subset**	0.0507	0.0638	**0.0495**
**Plant-life subset**	0.0469	0.0445	**0.0434**
**Rural subset**	0.0354	**0.0331**	0.0369
**Subset average**	0.0507	0.0435	**0.0396**
**Complete set**	0.0756	0.0388	**0.0358**

**Table 9:** The bi-directional translation outcomes of TaijiGNN on the ICVL database.

TaijiGNN Train(32 Samples):Test(169 Samples)					
**Metrics**	**SSIM**	**PSNR**	**rRMSE**	**RMSE**	**RMSE_INT**
**MSI to RGB**	1.000	54.994	0.004	0.002	0.492
**RGB to MSI**	0.999	44.665	0.029	0.007	1.700

**Table 10:** The RGB to MSI RMSE changes against the β variations.

β	PSNR	SSIM	RMSE (0–1)	RMSE (0–255)
0.01	20.149	0.6944	0.1073	27.368
0.1	33.383	0.9692	0.0247	6.302
0.5	33.821	0.9731	0.0232	5.915
1	33.683	0.9727	0.0234	6.010
2	33.782	0.9728	0.0234	5.957
3	33,767	0.9728	0.0232	5.966
CAVE dataset, train_epoch = 300, batch_size = 10,240				

**Table 11:** The MSI to RGB RMSE changes against the β variations.

β	PSNR	SSIM	RMSE (0–1)	RMSE (0–255)
0.01	16.672	0.7793	0.1569	40.013
0.1	38.261	0.9784	0.0136	3.467
0.5	46.318	0.9919	0.0054	1.381
1	46.273	0.9911	0.0056	1.423
2	50.557	0.9924	0.0034	0.875
3	50.955	0.9953	0.0032	0.809
CAVE dataset, train_epoch = 300, batch_size = 10,240				

## Data Availability

Not applicable.

## Appendix A. TaijiGNN Performance Summary

Table A1 shows the performance summary of TaijiGNN on the CPU and GPU. From the table, we can find that the GPU speed is about 10× faster than the CPU.

**Table A1:** The performance summary of TaijiGNN on the CPU and the GPU. (Batchsize = 10,240).

		CAVE (CPU)	CAVE (GPU)	ICVL (CPU)	ICVL (GPU)
**Training image size**		RGB (16, 512, 512, 3) MSI (16, 512, 512, 31)	RGB (16, 512, 512, 3) MSI (16, 512, 512, 31)	RGB (16, 512, 512, 3) MSI (16, 512, 512, 31)	RGB (16, 512, 512, 3) MSI (16, 512, 512, 31)
**Average one epoch** **training time (s)**		113	8	7	113
**Inferencing image size**		RGB (16, 512, 512, 3) MSI (16, 512, 512, 31)	RGB (16, 512, 512, 3) MSI (16, 512, 512, 31)	RGB (1, 1392, 1300, 3) MSI (1, 1392, 1300, 31)	RGB (1, 1392, 1300, 3) MSI (1, 1392, 1300, 31)
**Average image** **inferencing time (s)**	RGB2MSI	9.5	1.1	4.1	0.4
	MSI2RGB	9.2	1.1	4.1	0.4
